# Inflammation Triggered by SARS-CoV-2 and ACE2 Augment Drives Multiple Organ Failure of Severe COVID-19: Molecular Mechanisms and Implications

**DOI:** 10.1007/s10753-020-01337-3

**Published:** 2020-10-08

**Authors:** Masae Iwasaki, Junichi Saito, Hailin Zhao, Atsuhiro Sakamoto, Kazuyoshi Hirota, Daqing Ma

**Affiliations:** 1 Division of Anaesthetics, Pain Medicine and Intensive Care, Department of Surgery and Cancer, Faculty of Medicine, Imperial College London, Chelsea and Westminster Hospital, London, UK; 2 Department of Anesthesiology and Pain Medicine, Graduate School of Medicine, Nippon Medical School, Tokyo, Japan; 3 Department of Anesthesiology, Hirosaki University Graduate School of Medicine, Hirosaki, Aomori Japan

**Keywords:** SARS-CoV-2, COVID-19, angiotensin-converting enzyme 2, renin-angiotensin system, cytokine storm, multiple organ failure

## Abstract

The widespread occurrence of severe acute respiratory syndrome coronavirus 2 (SARS-CoV-2) has led to a pandemic of coronavirus disease 2019 (COVID-19). The S spike protein of SARS-CoV-2 binds with angiotensin-converting enzyme 2 (ACE2) as a functional “receptor” and then enters into host cells to replicate and damage host cells and organs. ACE2 plays a pivotal role in the inflammation, and its downregulation may aggravate COVID-19 *via* the renin-angiotensin system, including by promoting pathological changes in lung injury and involving inflammatory responses. Severe patients of COVID-19 often develop acute respiratory distress syndrome and multiple organ dysfunction/failure with high mortality that may be closely related to the hyper-proinflammatory status called the “cytokine storm.” Massive cytokines including interleukin-6, nuclear factor kappa B (NFκB), and tumor necrosis factor alpha (TNFα) released from SARS-CoV-2-infected macrophages and monocytes lead inflammation-derived injurious cascades causing multi-organ injury/failure. This review summarizes the current evidence and understanding of the underlying mechanisms of SARS-CoV-2, ACE2 and inflammation co-mediated multi-organ injury or failure in COVID-19 patients.

## INTRODUCTION

Coronavirus disease-2019 (COVID-19) is a highly transmissible disease caused by severe acute respiratory syndrome coronavirus-2 (SARS-CoV-2). The majority of COVID-19 patients have only mild symptoms and do not need hospitalization [48, 179]. Compared to the outbreaks of severe acute respiratory syndrome (SARS; 2002–2003; 774 deaths/8096 cases, case fatality rate (CFR) 9.6%) and Middle East respiratory syndrome (MERS; 2012–ongoing; 858 deaths/2494 cases, CFR 34.4%), the CFR of COVID-19 is relatively low (333,401 deaths/5,103,006 cases, CFR 6.5%) (recorded on May 23, 2020), but SARS-CoV-2 infection has caused the worst death toll worldwide [179]. Indeed, a certain percentage of COVID-19 patients (approx. 5%) were severe cases and often developed acute respiratory distress syndrome (ARDS), systemic inflammatory response syndrome (SIRS), and multiple organ dysfunction/failure with high mortality (CFR 49%) [179]. It has been suggested that respiratory failure associated with ARDS is the leading cause of death in COVID-19 cases [132].

The severity of COVID-19 is correlated with the levels of interleukin (IL)-6, C-reactive protein (CRP), and other proinflammatory cytokines [132, 175]. It was also found that the combination of d-dimer and IL-6 can be used to distinguish severe COVID-19 patients (area under the receiver operating characteristic [ROC] curve: 0.840) [39], suggesting that the high mortality rate of COVID-19 might be caused by the subsequent SIRS induced by a “cytokine storm.” It has been indicated that early interventions to attenuate this cytokine storm may improve the clinical outcomes of severe cases of COVID-19 [185].

Angiotensin-converting enzyme (ACE) 2 is a common binding site (“receptor”) for both SARS-CoV and SARS-CoV-2 [60]. The SARS-CoV-2 entry into host cells begins with its viral spike (S) protein binding to the host cell’s surface transmembrane ACE2, followed by a downregulation of membrane ACE2 expression [74]. The normal level of ACE2 is important to protect vital organs; However, as demonstrated in the models of acute lung injury (ALI) and ARDS [53, 62], the abnormal ACE2 levels were suggested to aggravate COVID-19 *via* the renin-angiotensin system (RAS), including promoting pathological changes in ALI [62] and being involved in inflammatory and fibrotic responses [141]. ACE2 may thus be a key disease mediator in the pathogenesis of COVID-19. Indeed, a recent publication indicated that higher ACE2 concentrations led to increased vulnerability to SARS-CoV-2 infection in men compared to women, and that this finding was also associated with the higher incidence and fatality rate of COVID-19 in men [133]. This review aims to summarize the multi-organ injuries and failure and the underlying mechanisms of COVID-19 that are associated with SARS-CoV-2 infection *via* ACE2 entry route and beyond.

## THE PHYSIOLOGY OF THE RENIN-ANGIOTENSIN SYSTEM AND ACE2

### The Systemic “Classic” Functions of RAS

**Fig. 1:**
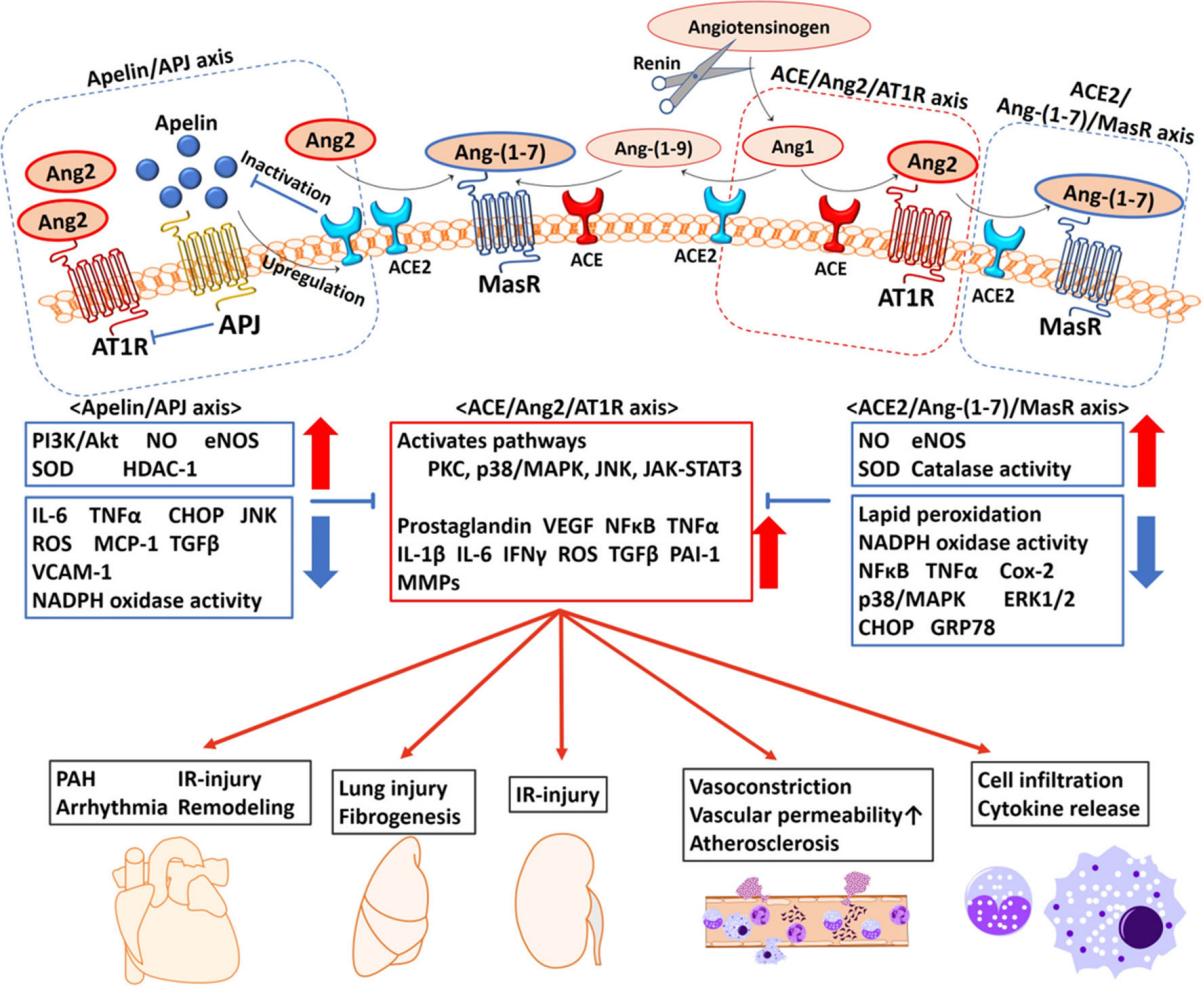
The enzymatic cascade and the apelin/APJ axis in the renin-angiotensin system (RAS). Multiple biological effects of RAS are mediated by Ang2, Ang-(1-7), and apelin. Ang2 is a central regulator of the inflammatory response, mainly through AT1R. As a proinflammatory modulator, Ang2 interacts on both immune cells and tissue-resident cells. The activated synthesis of Ang2 from tissue-resident cells enhances vascular permeability by promoting the productions of proinflammatory factors including prostaglandins, VEGF, NFκB, TNFα, IL-1β, IL-6, and IFNγ *via* the activation of several pathways. Ang2 also recruits immune cells into the injury site(s) and enhances the inflammatory response by stimulating the production of cytokines/chemokines, resulting in fibrosis and tissue injury. ACE2 inactivates Ang2 into mainly Ang-(1-7), and thus the ACE2/Ang-(1-7)/MasR axis is the negative regulatory axis against the ACE/Ang2/AT1R axis in the RAS. Apelin antagonizes the ACE/Ang2/AT1R axis through negative feedback by ACE2 upregulation. In addition, the molecular interaction between AT1R and APJ suppresses the activity of AT1R. The activation of the ACE2/Ang-(1-7)/MasR axis and the apelin/APJ axis has shown an organoprotective effect. ACE, angiotensin-converting enzyme; Ang1, angiotensin 1; Ang2, angiotensin 2; AT1R, angiotensin 1 receptor; CHOP, CCAAT/enhancer-binding protein homologous protein; Cox-2, cyclooxygenase-2; eNOS, endothelial nitric oxide synthase; ERK1/2, extracellular signal-regulated kinase; GRP78, glucose-regulated protein 78; HDAC-1, histone deacetylase-1; IFNγ, interferon gamma; IL-1β, interleukin-1 beta; IL-6, interleukin-6; IR-injury, ischemia reperfusion injury; JAK-STAT3, Janus kinase-signal transducer and activator of transcription system; JNK, C-jun-N-terminal kinase; MAPK, mitogen-activated protein kinase; MasR, Mas receptor; MCP-1, reactive oxygen species; MMP, matrix metalloproteinase; NADPH, nicotinamide adenine dinucleotide phosphate; NFκB, nuclear factor kappa-light-chain-enhancer of activated B cells; NO, nitric oxide; PAH, pulmonary artery hypertension; PAI-1, plasminogen activator inhibitor-1; PI3K/Akt, phosphoinositide 3-kinase/protein kinase B; PKC, protein kinase C; ROS, reactive oxygen species; SOD, superoxide dismutase; TGFβ, transforming growth factor-beta; TNFα, tumor necrosis factor alpha; VCAM-1, vascular cell adhesion molecule-1; VEGF, vascular endothelial cell growth factor.

As illustrated in Fig. 1, ACE2 is a key enzyme in the RAS, with multiple physiological functions [60]. The RAS is known as a major regulator of a wide range of physiology and pathophysiology [38, 118] such as the mediation of fluid/electrolyte homeostasis and the maintenance of vascular tone *via* angiotensin type 1 receptor (AT1R) in vital organs (kidneys, vascular smooth muscle, lung, heart, brain, adrenals, pituitary gland, and liver) [53]. For example, when the renal blood flow is reduced, the kidneys’ juxtaglomerular cells secrete renin directly into the blood circulation. This secreted renin converts angiotensinogen released by the liver to be angiotensin1 (Ang1), which is then converted to be angiotensin2 (Ang2) by ACE in pulmonary vascular endothelial cells [117, 118, 123]. Ang2 plays a central role in the RAS by acting on the angiotensin type 2 receptors AT1R and AT2R.

### The Local “Tissue” and “Intracellular” RAS

In addition to the systemic RAS, the physiological functions of RAS can been seen at the local “tissue” and even “intracellular” levels in the heart, lung, brain, kidney, liver, intestine and other digestive system [77, 82, 117]. The tissue RAS is involved mainly in cardiovascular regulation and inflammatory processes such as vascular permeability and tone [117] and cell apoptosis [25], growth [26], migration [26], and differentiation [27, 94]. The intracellular RAS is involved in the intracellular signaling pathways; Ang2 stimulates the production of reactive oxygen species (ROS) and nuclear factor kappa B (NFκB) *via* AT1R and the phosphatidylinositol-4,5-bisphosphate 3-kinase/protein kinase B (PI3K/Akt) pathway, leading to increases of proinflammatory cytokines such as IL-6, chemokines, and adhesion molecules in tissue-resident cells in the amplifying inflammatory cycle [38].

### ACE2, Angiotensin-(1-7), and the Mas Receptor Axis: Anti-inflammatory Property

ACE2 exists in two forms, the full-length transmembrane ACE2 (ACE2) and the soluble ACE2 (sACE2). sACE2 is cleaved from ACE2 by ADAM17 (a disintegrin and metallopeptidase domain 17) and then released into the extracellular environment [79]. ACE2 is the predominant enzyme regulating the ACE2/Ang-(1-7)/Mas receptor (MasR) axis. The function of sACE2 remains unclear. ACE is a close homolog of ACE2 with a 42% identical sequence in the catalytic domains, which function in an opposite manner to ACE for balancing [162]. ACE2 inactivates Ang2 into mainly Ang-(1-7) and converts Ang1 to Ang-(1-9), whereas ACE inactivates Ang1 into mainly Ang2 and converts Ang(1-9) to Ang-(1-7) [162]. Thus, the ACE2/Ang-(1-7)/MasR axis is the negative regulatory axis against the ACE/Ang2/AT1R axis in the RAS.

Ang-(1-7) exerts anti-inflammatory effects *via* MasR and G proteins. ACE2/Ang-(1-7)/MasR attenuated the local and systemic inflammation reported in various experimental models including sepsis [158], acute lung injury [71], atherosclerosis [189], and chronic kidney disease in mice [31]. Ang-(1-7) also inhibited the release of inflammatory cytokines (IL-6 and tumor necrosis factor alpha [TNFα]) from macrophages that was induced by lipopolysaccharide (LPS) in endotoxemic mice [142, 143]. Ang-(1-7) also bind to both AT1R and AT2R at high concentrations [163]. In a rheumatoid arthritis model, the ACE2/Ang-(1-7)/AT2R axis also provided an anti-inflammatory response by reducing the gene expressions of IL-1β and IL-6 and activating NFκB [157]. Ang-(1-7) inhibited AT1R in a non-competitive manner [120].

In general, the ACE2/Ang-(1-7)/A2R/MasR axis is considered to be a multi-organ protector opposing the ACE/Ang2/AT1R axis. The function of sACE2 remains unclear, but high levels of sACE2 were found in patients with SARS [65], type 1 or type 2 diabetes [44], hypertension [167], heart failure [1], and chronic kidney diseases [1], suggesting that increased sACE2 may act in a protective manner to counteract the adverse effects of Ang2. SARS-CoV-infected wild-type mice had significantly reduced ACE2 expressions in the lung [74] and heart [110].

Pulmonary infectious mice showed an ACE2-dependent myocardial injury with a remarkable decrease of ACE2 expression, indicating a key role of ACE2 in mediating SARS-CoV infection in the heart [110]. Further, *ace2* knockout mice exhibited enhanced vascular permeability, increased lung edema, induced neutrophil accumulation in the lung, and worsened lung function [62]. Notably, treatment with recombinant human ACE2 protein (rhACE2) improved the symptoms of acid aspiration- or LPS-induced acute lung injury in mice [62].

### Interaction with ACE2 and the Apelin/APJ Axis: Organ Protection

Apelin is another substrate of ACE2 and an endogenous peptide ligand to the G protein–coupled receptor, the APJ [75]. Apelin is expressed predominantly in the endothelium and acts locally *via* endocrine signaling to activate the APJ, which is expressed on the surface of myocardial cells and endothelial cells [18, 72]. The structure of apelin is similar to that of Ang2 but without binding affinity to AT1R [153]. Apelin-13, a predominant isoform of apelin, antagonizes the ACE/Ang2/AT1R axis through negative feedback by ACE2 upregulation [134]. In addition, the molecular interaction between AT1R and APJ suppresses the activity of AT1R [24]. The apelin/APJ axis reduces vascular tone, decreases blood pressure, regulates fluid homeostasis, improves cardiac contractility [75], and protects against heart [124] and lung injury [35]. In both oleic acid- and LPS-induced ARDS models, treatment with apelin-13 after injury attenuated the lung injury and improved oxygenation [35].

### ACE, Ang2, and the AT1R Axis: Proinflammatory Property

In contrast to the ACE2/Ang-(1-7)/MasR axis, the ACE/Ang2/AT1R axis accelerates the inflammatory response. In this axis, Ang2 is a central regulator of the inflammatory response through specific cell surface receptors (mainly AT1R). As a proinflammatory modulator, Ang2 interacts with both immune cells (neutrophils, mononuclear cells, T cells, and B cells) and tissue-resident cells. The activated Ang2 synthesis from tissue-resident cells enhances vascular permeability by promoting the productions of proinflammatory factors including prostaglandins, vascular endothelial cell growth factor (VEGF) [173], NFκB, TNFα, IL-1β, IL-6, and interferon gamma (IFNγ) [38].

Ang2 also recruits immune cell infiltration into the injury site(s) and enhances the inflammatory response by stimulating the production of cytokines/chemokines. For example, Ang2 induced the proliferation of splenic lymphocytes *via* the activation of AT1R on immune cells [106]. In addition, Ang2 upregulates the expression of Toll-like receptor 4 (TLR4), stimulates NFκB signaling, and induces the expressions of CD40, TNFα, IL-6, and MMP9 (matrix metallopeptidases) [64, 178].

TNFα is a key proinflammatory cytokine that acts to cross-link inflammation and the RAS, and its extracellular domain shedding and activation are driven by the ADAM17 on the cell surface [13]. AT1R activation by Ang2 binding phosphonates ADAM17 *via* the intracellular mitogen-activated protein kinase (p38/MAPK) cellular signaling pathway facilitates the cleavage of ACE2 and tends to the ACE/Ang2/AT1R axis, leading to a positive feedback mechanism in the proinflammatory response [116].

### Epithelial and Endothelial Cells: Receptors of SARS-CoV-2

ACE2 was identified as the binding “receptor” of SARS-CoV and SARS-CoV-2. ACE2 is expressed predominantly in the epithelial cells of the lung and intestine [54], suggesting that these organs may be the primary infected sites of SARS-CoV-2. ACE2 is also present in arterial and venous endothelial cells [54]. These distributions of ACE2 are very likely associated with the characteristics of COVID-19: respiratory failure, colitis, microvascular injury, and inflammation.

In COVID-19, the airways and lungs are the main injured organs, and respiratory failure is the leading cause of death [132]. A high expression of ACE2 was identified in alveolar epithelial cells of the lungs of COVID-19 patients [54, 121, 196]. The profiling of the expression of *ace2* RNA in healthy human lung tissue revealed that 83% of the ACE2-expressing cells were alveolar epithelial cells [190]. Multiple ESCRT (endosomal sorting complexes required for transport) machinery genes (including CHMP3, CHMP5, CHMP1A, and VPS37B genes) were shown to be related to virus budding and release [190]. These findings indicate that aside from ACE2, other intracellular mechanisms in alveolar epithelial cells facilitate virus replication in the lungs.

ACE2 is also highly expressed in intestinal epithelial cells [54, 121, 196], and the intestine may be another potential viral target organ. In an *in vitro* study using human small intestinal organoids, enterocytes were infected by SARS-CoV or SARS-CoV-2; these small infection clusters had spread throughout the entire organoids at 60 h post-infection, and the levels of infectious virus particles and viral RNA were significantly increased in both viruses [80]. These data suggested that the intestine might also be an entry site for SARS-CoV-2, and the virus’ ability to be transmitted *via* the mouth/food intake is thus worth investigating.

Severe cases of COVID-19 were frequently characterized by coagulopathy and microvascular injury, leading to organ dysfunction [151, 191]. The expression of ACE2 in vascular endothelial cells probably contributes to the pathophysiology of microcirculatory pathological changes. The histopathology from three cases of COVID-19 revealed a direct SARS-CoV-2 infection to the endothelial cells leading to diffuse endothelial inflammation [160], which was similar to an earlier report in SARS-CoV-infected patients [184]. The recruitment of immune cells resulted in widespread endothelial dysfunction due to apoptosis [160]. It is thus very likely that microvascular inflammation and dysfunction contribute to the clinical sequelae of multiple organ failure in COVID-19 patients.

## THE PATHOLOGY OF THE CYTOKINE STORM

### The Macro-mechanism of the Cytokine Storm in General Inflammation

The cytokine storm begins from a local site of inflammation and then spreads throughout the entire body *via* an overproduction of inflammatory cytokines and chemokines released by both immune and non-immune cells. These events were directly correlated with organ injury and poor prognosis [101].

Innate immunity was suggested to play a central role in the pathology of this cytokine storm through pattern recognition receptors (PRRs) [23] such as the TLRs, the Nod-like receptors, the RIG-like receptors, and the C-type lectin receptors [68]. Leukocyte activation is mediated by the PRRs, which bind to a wide variety of molecules including pathogen-associated molecular patterns (PAMPs) and the damage-associated molecular patterns (DAMPs) released from infected cells [23, 146, 149].

In the early stage of infectious inflammation, IL-6 is rapidly produced by monocytes/macrophages stimulated by TLRs [136]. The membrane IL-6 receptor (mIL-6R) is expressed only in some immune cells, but the soluble IL-6 receptor (sIL-6R) is widely present in both immune and non-immune cells [131]. IL-6 induces various actions to activate glycoprotein 130 (gp130, which is expressed in most cells) by the IL-6 receptor complex (the details of the mechanism of action are described below) [98]. IL-6 is an essential cytokine for maintaining homeostasis in the body, and when stress is removed, the synthesis of IL-6 ceases but excess IL-6 production causes chronic inflammatory diseases and a severe systemic inflammatory response, *i.e.*, the cytokine storm [101].

### The Micro-mechanism of the Cytokine Storm in COVID-19

**Fig. 2:**
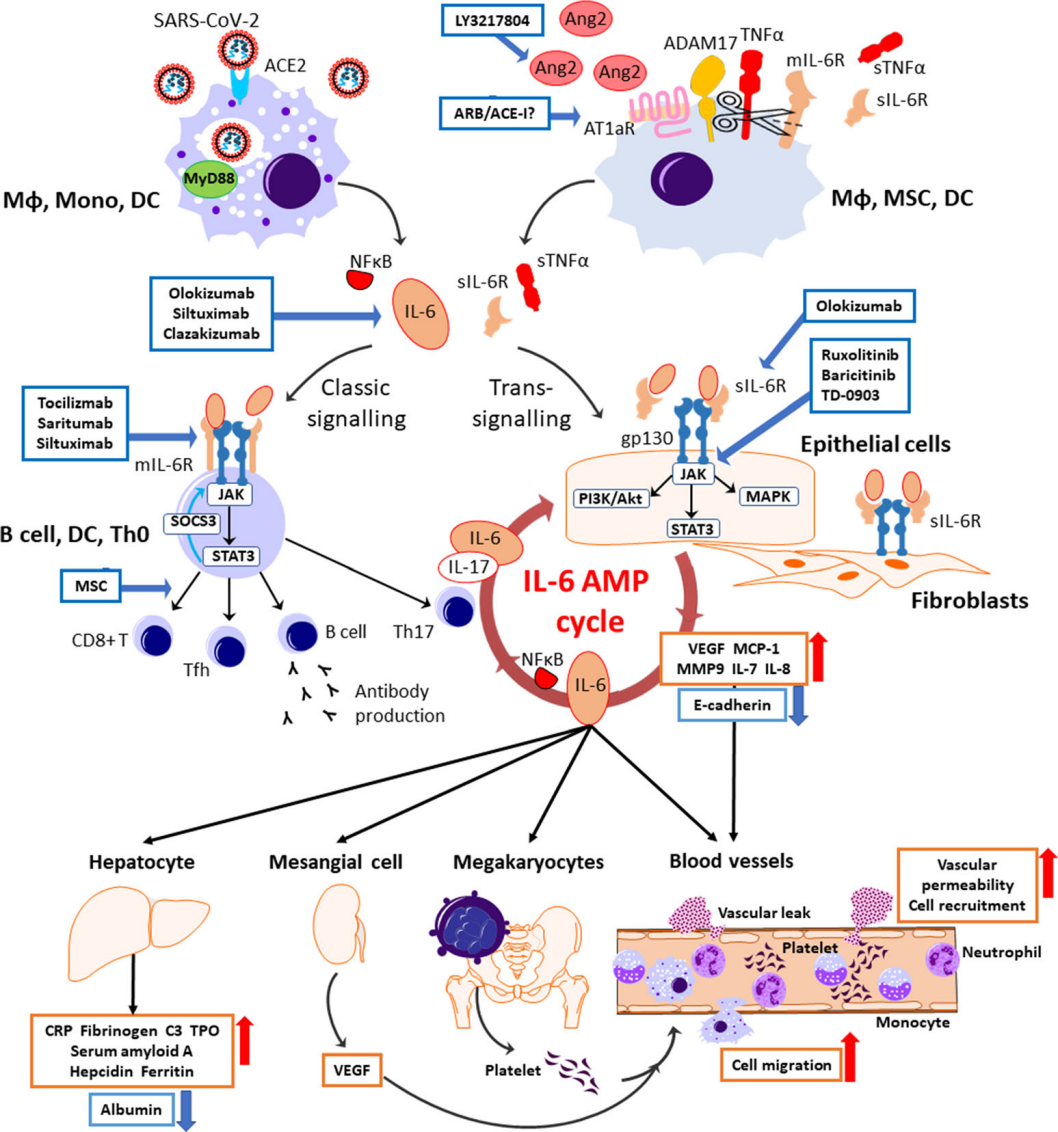
The therapeutics under clinical trials targeting the cytokine storm in SARS-CoV-2 infection. SARS-CoV-2 attaches to ACE2 and enters the host cell. Viral components are recognized by the MyD88 pathway in the endosome, leading to the releases of IL-6 and NFκB from immune cells including macrophages, monocytes, and dendrites. The possession of ACE2 by the virus causes the accumulation of Ang2 in the extracellular space. After the activation of AT1aR by excess Ang2 binding, sIL-6R is produced from the shedding of mIL-6R by ADAM10 and ADAM17 with a release of sTNFα from macrophages, mesenchymal stem cells (MSCs), and dendritic cells (DCs). IL-6 binds to the target cells *via* two signaling pathways: classic signaling only for specific immune cells, and trans-signaling for any cells including the immune cells, epithelial cells, and fibroblasts. In the classic signaling pathway, IL-6 binds to mIL-6R on the immune cells and activates B cells or differentiates CD8+ T cells, helper T cells, and Th17 cells, which triggers an anti-inflammation response. There is a negative feedback mechanism to the JAK-STAT pathway by SOCS3. In trans-signaling, IL-6/sIL-6R complex can bind to gp130 following the release of proinflammatory cytokines and IL-6 *via* three intracellular pathways without SOCS3 negative feedback. Since gp130 is highly expressed on almost all types of cells including the immune cells, sIL-6R shedding by ADAM17 provokes a surge of IL-surge mostly *via* trans-signaling. IL-6 stimulates the production of IL-6 and IL-17α from Th17 cells, resulting an IL-6 burst in its amplification cycle. The proinflammation cytokines increase vascular permeability and cell migration, enhancing the inflammation response. IL-6 also stimulates megakaryocytes, renal mesangial cells, and hepatocytes with the subsequent inflammatory response and vital organ injury. ARB/ACE-I, angiotensin receptor blocker/ACE2 inhibitor; AT1aR, angiotensin receptor subtype 1a; C3, complement component 3; E-cadherin, epithelial cadherin; gp130, glycoprotein 130; IL, interleukin; JAK, Janus kinase; MAPK, mitogen-activated protein kinase; MCP-1, monocyte chemoattractant protein 1; mIL-6R, membrane interleukin 6 receptor; MMP9, matrix metallopeptidase 9; MyD88, myeloid differentiation primary response 88; NFκB, nuclear factor kappa-light-chain-enhancer of activated B cells; NFκB, nuclear factor kappa B; PI3K/Akt, phosphoinositide-3-kinase/protein Kinase B; SARS-CoV-2, severe acute respiratory syndrome coronavirus 2; sIL-6R, soluble interleukin 6 receptor; SOCS3, the suppressor of cytokine signaling-3; STAT3, signal transducers and activators of transcription; sTNFα, soluble tumor necrosis factor alpha; Tfh, follicular helper T cell; Th0, naive T cell; Th17, T helper 17 cell; TMPRSS2, transmembrane protease serine 2; TNFα, tumor necrosis factor alpha; TPO, thrombopoietin; VEGF, vascular endothelial growth factor.

SARS-CoV-2 binds directly to the host cells’ surface ACE2. The virus cleavage in the host endosome activates NFκB *via* TLRs and the MyD88 (myeloid differentiation primary response 88) pathway [168], which stimulates IL-6 protein transcription in the host immune cells. Once ACE2 is occupied by the virus, free Ang2 accumulates in the plasma due to a lack of degradation by ACE2. The mIL-6R exists only on immune cells such as T cells, monocytes, macrophages, activated B cells, neutrophils, and osteoclasts [131], where the classic IL-6 signaling occurs following the anti-inflammatory bioreaction. After the A2R activation by excess Ang2 binding, sIL-6R is derived from the shedding of mIL-6R by ADAM10 and ADAM17 [40]. In contrast, gp130 is expressed widely as another type of IL-6R on the surface of non-immune cells, and it can be activated by a trans-signaling of IL-6 and sIL-6R [130] or the trans-presentation of IL-6/mIL-6R complex on another immune cell [56] (Fig. 2).

The IL-6 trans-signaling leads to an immense effect due to the widespread presence of gp130 on both immune and non-immune cells and also due to the suppression of intracellular negative feedback by SOCS (suppressor of cytokine signaling) 3 *via* the Janus kinase-signal transducers and activators of transcription (JAK-STAT3) pathway in the immune cells [105]. When the IL-6/IL-6R/gp130 complex is formed, the IL-6 signal is transmitted *via* three intracellular signaling pathways (*i.e.*, the JAK-STAT [especially STAT3], RAS/MAPK, and PI3K/Akt pathways), producing proinflammatory cytokines such as NFκB, VEGF, MMP9, and IL-6. In addition, non-immune cells such as endothelial cells, smooth muscle cells, and fibroblasts are activated by NFκB and STAT3 and then release IL-7, which stimulates CD4-positive cells to release IL-6 and IL-17a, resulting in positive feedback to the non-immune cells in the IL-6 amplification cycle [103]. This IL-6 hyperactivation cycle induces the systemic status recognized clinically as the cytokine release syndrome, or cytokine storm.

### Organ Injury by the Cytokine Storm

The lungs are the organ that is most vulnerable to the above-described cytokine storm, and ARDS is a common, fatal condition that follows a cytokine storm in the alveolar environment and systemic circulation, induced by SARS-CoV, MERS-CoV, and severe influenza as well as SARS-CoV-2 [159, 171]. An acute mononuclear/neutrophilic inflammatory response induced ARDS, followed by a chronic fibroproliferative phase with progressive collagen deposition in the lung. This may be explained by the findings that pulmonary endothelial cells [155, 156] and epithelial cells [78] have critical roles in both the promotion of cytokine amplification and the innate immune cell recruitment by cytokine storm during viral infection.

After N1H1 influenza infection, pulmonary endothelial cells secreted cytokines (IL-6, IFNα, IFNγ, and TNFα) and chemokines (CCL-2) and recruited innate immune cells (macrophages and natural killer cells) but not lymphocytes [155]. Due to the injurious effects of these proinflammatory cytokines and chemokines, vascular permeability increases, and fluid and blood cells leak into the alveoli, resulting in dyspnea and even respiratory failure.

## THE MECHANISMS UNDERLYING SARS-COV-2 INFECTION

### SARS-CoV and SARS-CoV-2

SARS-CoV-2 is a single-stranded RNA virus with a high similarity to SARS-CoV, sharing 80% genome sequence identity [49, 164, 174, 187] and 90% sequence identity in their N-terminals of the receptor-binding domain and similarity to other essential enzymes [109, 174]. Both viruses have a nucleocapsid inside with two types of spike proteins (S1 and S2) on the viral surface. S1 is involved in the attachment of the virus to host cells and is considered to be a target of neutralizing antibodies [43]. S2 is involved in cell membrane fusion [34] and is a potential target of fusion inhibitors. Based on clinical analyses of the basic reproductive number (R0), SARS-CoV-2 (R0 2.2–2.7 [139, 177]) appears to have infectivity that is comparable to that of SARS-CoV (R0 1.5–3.4 [22, 86, 128]). In addition, the latest genome analyses demonstrated that SARS-CoV-2 has its own sequence for furin cleavage [28, 164].

### ACE2: a Functional “Receptor” of SARS-CoV-2

**Fig. 3:**
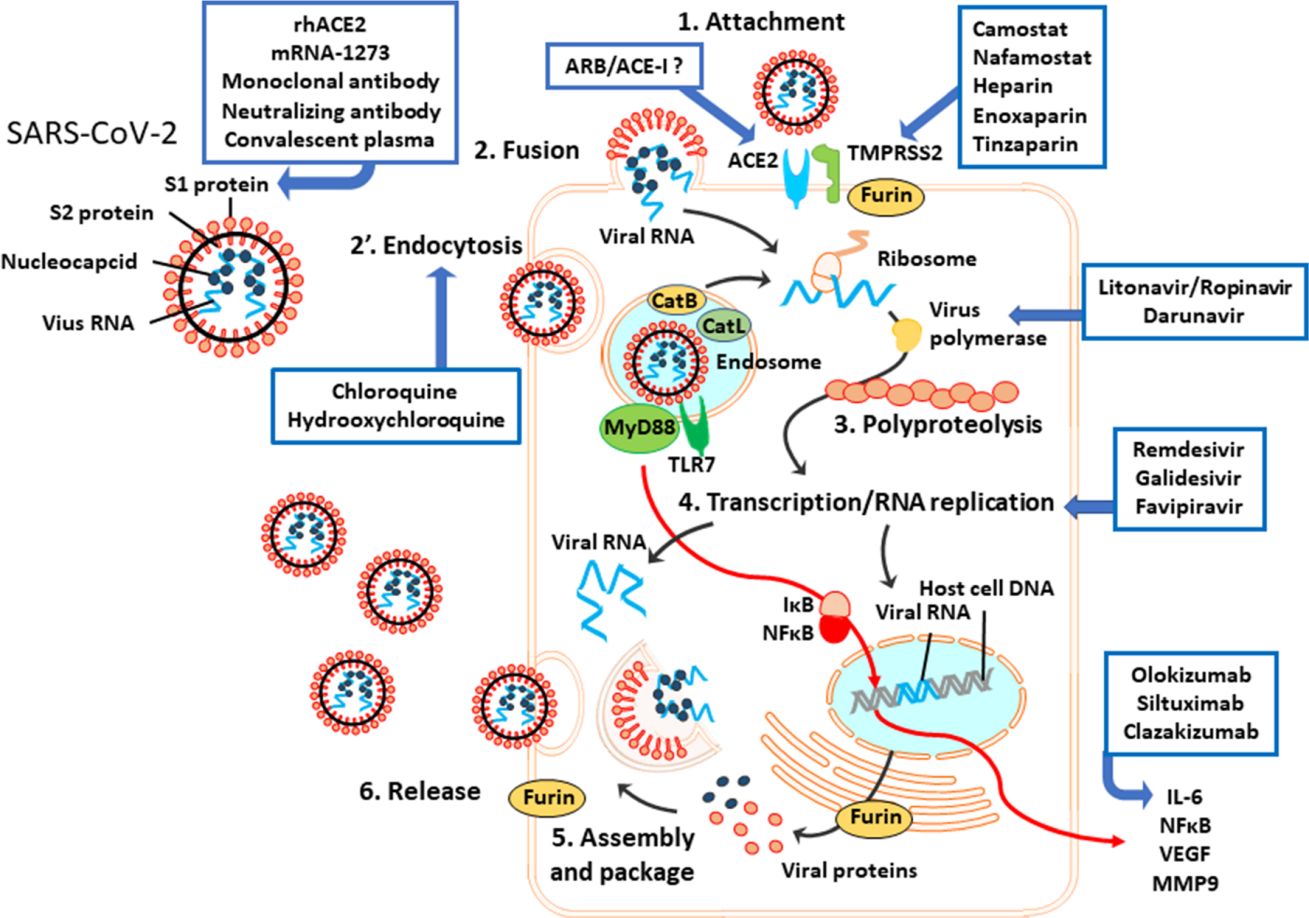
The putative mechanisms of SARS-CoV-2 infection. SARS-CoV-2 has two surface proteins and single-strand RNA with nucleocapsid proteins. S1 protein binds to the host ACE2 by cleavage with TMPRSS2 and furin. After this attachment, the virus enters the host cell *via* fusion or endocytosis. In the endosome, cathepsins B and L activate the S2 protein of the virus for membrane fusion. The virus components are recognized by TLR7, leading to the release of proinflammation cytokines (IL-6, NFκB, VEGF, and MMP9) *via* the MyD88 pathway in immune cells such as macrophages, monocytes, and dendritic cells. Once the virus RNA is released in the host cytoplasm, the virus polypeptide chain with ribosome translation is processed in the replication/transcription complex by virus RNA polymerase. Replicated virus RNA and proteins are assembled and packed with the host membrane in the host cytoplasm. Virus is released from the cell by exocytosis or the host cell’s burst. ARB/ACE-I, angiotensin receptor blocker/ACE inhibitor; Cat L, cathepsin L; CatB, cathepsin B; IκB, inhibitory proteins of κB family; MMP9, matrix metallopeptidase 9; MyD88, myeloid differentiation primary response 88; NFκB, nuclear factor kappa-light-chain-enhancer of activated B cells; rhACE2, recombinant human ACE2 protein; TLR7, Toll-like receptor 7; TMPRSS2, transmembrane protease serine 2.

SARS-CoV-2 can enter host cells *via* two mechanisms: “endosome attachment fusion” and “direct-attachment fusion” (Fig. 3). After virus attachment, the S1 protein of SARS-CoV-2 binds to ACE2 on the host cell membrane to enter the cell [83] after the activation of S1 protein (“cleavage”) by host proteinase “priming.” The viral S1 protein priming depends on transmembrane protease serine 2 (TMPRSS2) and other host proteases including the endosomal cysteine proteases cathepsins B and L (CatB/L) [60]. TMPRSS2 is a co-factor of ACE2 and is co-expressed in type II pneumocytes with ACE2 [9, 45, 145, 195], bronchial transient secretory cells [88], and nasal secretory cells [195], which are known as enriched sources of SARS-CoV-2 to be detected clinically [193].

The co-expression of ACE2 and TMPRSS2 was reported in type 1 pneumocytes [195], alveolar macrophages [9, 192], lymphocytes [9], smooth muscle cells and enterocytes in the gastrointestinal tract [9, 195], vessel smooth muscle cells [9], cardiomyocytes [9, 192], hepatocytes [169], kidney cells [87], proximal tubule cells [114], and neurons [112], all of which are in line with the common clinical symptoms of COVID-19 [15, 19, 61, 151, 166, 183, 191, 197]. Single-cell RNA profiling revealed that male gender, advanced age, and smoking habit can increase the co-expression of ACE2 and TMPRESS2 [104], and these are known as clinical risk factors for severe COVID-19 [61, 63, 151, 166, 175, 191, 194].

Genome analyses showed that SARS-CoV-2 has a unique sequence for a furin cleavage site [28, 164], which may determine the viral affinity to host cells [70, 165]. Furin, a peptidase that is also known as PACE (paired basic amino acid cleaving enzyme), may also be involved in this process. Recent research revealed that furin cleaved the S1/S2 proteins of SARS-CoV-2 and was essential for virus fusion with the host membrane [59]. It has been estimated that compared to SARS-CoV, SARS-CoV-2 has 10–20-fold higher affinity to host membrane ACE2 [174], possibly due to its own FURIN sequence [34]. Furin is highly expressed in lung tissue [4, 28] and it was observed that 43% of furin+ bronchial transient secretory cells were co-expressed with TMPRESS2 [88]. The triple expression of ACE2, TMPRSS2, and furin was detected in lung macrophages, kidney, adrenal stromal cells, intestine endocytes [192], and nasal epithelial cells [176], strongly suggesting that those organs are susceptible to SARS-CoV-2 infection.

### SARS-CoV-2 Entry Associated with Host ACE2, TMPRSS2, and Furin

Once the viral S1 protein attaches to the host cell membrane’s ACE2, the virus enters the cell by host endocytosis, which is dependent on the host cell’s clathrin [20, 60]. Viral S2 protein is activated by the proteolysis cleavage with the host cell’s CatB/L in endosomes [7, 99, 140] or with TMPRSS2, CatB/L, and furin in the extracellular space [60], inducing viral envelope fusion to the host membrane and the release of the virus component.

CatB was reported to be co-expressed in > 70–90% of human ACE2+ nasal secretory cells [144]. At the same time, the virus cleavage *via* TLR7 and the MyD88 pathway activates the production of NFκB, which stimulates IL-6 protein transcription in the host immune cells [168]. The replication-transcription complex processes the viral genome replication in the cytoplasm, and the transcription of viral proteins is processed in the host nucleus separately. The replicated viral RNA and protein are then assembled in the host cytoplasm, and the virus envelope is modified by the host components. Replicated virus buds from the host cells by exocytosis or the bursting of the host cell.

### SARS-CoV-2 Infection Induces a Cytokine Storm with an Interaction with the RAS

Simultaneously, the ACE2 possession by SARS-CoV-2 induces accumulations of sACE2 in the blood and urine [12], with the subsequent cytokine storm including the release of IL-6 from the host macrophage [14, 147] as described above. These events are closely linked to the worsening of lymphocytopenia [30], hypercoagulation [97], higher mortality [175], and poor clinical outcomes [10, 11, 61, 122]. It was shown that in SARS-CoV infection, the activation of A2R (mostly AT1aR) by Ang2 binding stimulates ADAM17 to promote the shedding of ACE2 into the extracellular space (which increases the uptake of SARS-CoV into cells [51, 52]) and leads to the direct cleavage of SARS-CoV S protein and the induction of the release of TNFα/IL-6 [58]. In addition, AT1aR and ADAM17 are co-expressed on various cell types such as endothelial cells [170] and vascular smooth muscle cells [108], which were reported to be involved in pathological lesions in COVID-19 patients [15, 61, 67, 166, 179].

In summary, SARS-CoV-2 entry is facilitated by the host cells’ ACE2, TMPRSS2, and furin, inducing the activation of A2R and the cytokine storm starting with IL-6 release. Insights into the co-expression of ACE2, TMPRSS2, and furin may help to identify the organs that are vulnerable to SARS-CoV-2 infection and may contribute to the development of potential therapeutic strategies.

## MULTI-ORGAN DYSFUNCTION/FAILURE

### Respiratory Failure/ARDS

As noted above, the lungs are the organ most vulnerable to SARS-CoV-2, and importantly, respiratory failure associated with ARDS is the leading cause of death in individuals with COVID-19, contributing to 86% of the deaths [132]. In the severe or critical cases in intensive care units (ICUs), 53–95% of the patients developed ARDS [2, 183, 197]. Among those, 2–11% of the patients with ARDS required extracorporeal membrane oxygenation support, and the survival rate of those patients was quite low [61, 166, 183, 191]. The onset time of ARDS related to COVID-19 was 8–12 days [61, 166, 191], which is not consistent with the ARDS Berlin criteria [37], but histological examinations revealed that the pathophysiological features are similar to the common ARDS [19].

### Myocardial Injury

Numerous studies revealed that acute cardiac injury was also a common complication of COVID-19 [2, 19, 50, 61, 138, 139, 166, 183, 191, 197] and was associated with poor outcomes among severe COVID-19 patients [50, 138, 139]. It has been proposed that this might be due to the development of heart failure [138, 191] and lethal arrhythmias [50]. Acute cardiac injury is defined as the elevation of cardiac troponin (high-sensitivity troponin I [hsTnI]) and/or troponin T ([TnT) to > 99th percentile alone, or a composite of troponin elevation and ECG or echocardiographic abnormalities [2, 19, 50, 61, 138, 139, 166, 183, 191, 197].

Two cohort studies showed that 19.7–27.8% of hospitalized COVID-19 patients exhibited myocardial injury as indicated by elevated hsTnI [139] and TnT [50]. The mortality rate of COVID-19 patients with cardiac injury was significantly higher than that of the patients without cardiac injury (51.2% *vs.* 4.5%, *p* < 0.001), and cardiac injury was an independent predictor of in-hospital mortality as well as ARDS [139]. The troponin level of COVID-19 patients was positively correlated with the level of NT-ProBNP (N-terminal-pro hormone B-type natriuretic peptide) l [50, 139], indicating that cardiac injury is associated with poor clinical outcomes among COVID-19 patients.

The mechanisms underlying cardiac injury in the context of COVID-19 remain to be investigated, but may be due to a direct injury from SARS-CoV-2 infection and then exacerbation by inflammatory responses. The expression of ACE2 on the myocardium [50] and vascular endothelial cells [54] provides a theoretical mechanism of the direct injury by SARS-COV-2 to the heart, with resultant myocarditis. Indeed, the viral RNA was detected in heart autopsies [172]. In addition, excess inflammation causes endothelial dysfunction and increases the prothrombotic activity of the blood, both of which may contribute to the formation of an occlusive thrombus, leading to acute coronary syndrome and myocardial injury.

### Acute Kidney Injury

The prevalence of acute kidney injury (AKI) varied depending on the severity of COVID-19 [2, 21, 48, 139, 183, 191, 197]. Two large observational studies showed that the prevalence of AKI was relatively low (0.5–5%) in hospitalized COVID-19 patients [21, 48]. Kidney functional abnormalities including proteinuria (43.9%) and hematuria (26.7%) were observed in many COVID-19 patients, and, importantly, the kidney abnormalities were independent predictors of in-hospital death: proteinuria of any degree, hematuria of any degree, elevated baseline blood urea nitrogen, serum creatinine, peak serum creatinine > 133 μmol/L, and AKI > stage 2 [21].

The mechanisms that underlie kidney injury/abnormality have been hypothesized to include both direct cytotoxic effects of SARS-CoV-2 itself and cytokine-mediated damage [21]. ACE2 was shown to express in glomerular parietal epithelial cells and epithelial basal cells in the kidneys and in kidney proximal tubules [121, 196]. SARS-CoV-2 enters the host cells *via* the binding of ACE2 on epithelial cells. Indeed, an evaluation of 12 autopsy cases revealed that six patients had viremia, and viral RNA was also detected in kidney tissues at concentrations exceeding viremia [172].

These data suggest that SARS-CoV-2 spreads through the bloodstream and directly injures the kidneys. The cytokine storm (a hyper-inflammatory status) might exert indirect effects on renal abnormalities such as hypoxia secondary due to respiratory failure, septic and cardiogenic shock, and rhabdomyolysis, all of which were reported in patients with severe influenza viral infection [76].

### Liver Injury

Several cohort studies reported that liver injury occurred in COVID-19 patients, and the liver injuries were described mainly as an increase in the level of alanine aminotransferase (ALT), aspartate aminotransferase (AST), or total bilirubin accompanied by slightly decreased albumin [2, 19, 50, 61, 175, 183, 191, 197]. Patient series with severe COVID-19 or non-survivors were more likely to have a higher prevalence of liver injury compared to mild cases and survivors [19, 61, 175, 191, 197].

In a retrospective study, liver injury was detected in 35.4% of non-ICU patients with COVID-19 [61]. Male patients and patients with high levels of white blood cells, neutrophils, and CRP and a greater extent of pulmonary lesions on computed tomography were more likely to have liver injury [61]. The expression of ACE2 was observed on endothelial cells of the liver and cholangiocytes as well as other vital organs [54, 121]. The study of 12 autopsy cases showed high SARS-CoV-2 RNA titers in the liver [172]. The liver biopsy of a COVID-19 patient revealed micro-vesicular steatosis and lobular and portal activity, suggesting that the injury was caused by either SARS-CoV-2 infection or drug-induced injury [19]. As cholangiocytes play pivotal roles in the initiation and regulation of immune responses and liver regeneration [3], it is possible that liver injury and bile duct injury are caused directly by viral infection.

### The Gastrointestinal Injury/Gut-Lung Axis

Digestive manifestations including lack of appetite, nausea, vomiting, diarrhea, abdominal pain, and gastrointestinal hemorrhage have been documented in patients with SARS-CoV-2 infection [19, 48, 66, 113, 166, 183, 188, 197]. COVID-19 patients with digestive manifestations were significantly more likely to require intensive care admission and to have ARDS compared to patients without gastrointestinal symptoms (6.8% *vs.* 2.1%, *p* = 0.034, and 6.8% *vs.* 2.1%, *p* = 0.034, respectively) [66], indicating an association between the presence of digestive symptoms and disease severity. The gut microbiota have been shown to affect lung health through an important crosstalk between the gut microbiota and the lungs, which is called the “gut-lung axis” [16]. The gut-lung axis is bidirectional, and microbial metabolites affect the lungs *via* the blood stream while inflammation in the lungs also affects the gut flora [16]. This raises the interesting possibility that SARS-CoV-2 may also affect the gut microbiota. Viral RNA has been detected in stool samples of COVID-19 patients [148, 180]. The SARS-CoV-2 itself may cause disorders of the intestinal flora, which may result in digestive symptoms and deterioration of patients with ARDS.

### Neurological Injuries

Neurological injuries including central nervous system (CNS) symptoms, peripheral nervous system (PNS) symptoms, and skeletal muscle injury symptoms have been observed in patients with mild to severe COVID-19 [8, 19, 81, 92, 102, 119, 186]. Two cohort studies [19, 81] reported high prevalence of olfactory (68–86%) and gustatory (71–88%) dysfunctions in COVID-19-positive patients, and olfactory dysfunction appeared before the other symptoms in 11.8% of the cases [19]. These two chemosensory dysfunctions were independently and strongly associated with COVID-19 positivity [19] and should be considered to be early signs of the disease.

A retrospective analysis of neurological manifestations of COVID-19 demonstrated that of 214 patients, 36.4% of the hospitalized patients had neurologic manifestations: CNS (24.8%), PNS (8.9%), and skeletal muscle (10.7%) injury [94]. In the groups of patients with CNS manifestations, neurologic disorders were more common in the severe patients compared to the non-severe patients; the disorders included acute cerebrovascular diseases (5.7% *vs.* 0.8%), impaired consciousness (14.8% *vs.* 2.4%), and neural skeletal muscle injury (19.3% *vs.* 4.8%) [92]. More severe neurological manifestations were reported in a few case reports [8, 102, 119]; for example, a 24-year-old male experienced a coma, seizures, and neck stiffness [102]. SARS-CoV-2 RNA was detected in his cerebrospinal fluid (CSF), and subsequent magnetic resonance imaging indicated right lateral ventriculitis and encephalitis mainly at the right mesial lobe and hippocampus, where SARS-CoV2-associated meningitis/encephalitis was diagnosed [102]. The mechanism may be a direct invasion of SARS-CoV-2, similar to SARS virus [181].

Brain tissues highly express ACE2 [138], and SARS-CoV-2 RNA was found in the CSF of the abovementioned COVID-19 patient [102] and in the patient’s brain autopsy [172]. As with other respiratory viruses, SARS-CoV-2 may enter the CNS through a hematogenous or retrograde neural route [92]. The latter route is supported by the finding that COVID-19 patients have a high prevalence of olfactory dysfunction. The results of the above-cited retrospective analysis suggested that the observed neurological symptoms may likely be associated with worse prognosis in COVID-19 [92].

A tissue profiling analysis showed that ACE2 is strongly expressed in the respiratory control center, the ventrolateral medulla, and the nucleus of the tractus solitarius [33, 111]. Other coronaviruses with structures that are similar to that of SARS-CoV-2 have been reported to cause the apoptosis of neurocytes in the respiratory center to a degree that is high enough to drive the lethal nervous-respiratory malfunction [32, 85, 93, 107]. Thus, the respiratory failure in COVID-19 might be caused in part by the invasion of SARS-CoV-2 to the neurons of the cardiorespiratory center in the ventrolateral medulla. The findings described above may explain why, in some COVID-19 patients, their respiratory function can be deteriorated very quickly and patients die suddenly.

### Microvascular Injuries

Hospitalized patients with severe COVID-19 had a prolonged prothrombin time (PT), a prolonged activated partial thromboplastin time (APTT), and elevated d-dimer [73, 127, 150, 151, 191]. Among those patients, some developed disseminated intravascular coagulopathy (DIC) [19, 150, 151]. A higher d-dimer level or higher sepsis-induced coagulopathy (*SIC*) score was associated with the severity of or death from COVID-19 [19, 29, 150, 191]. The clinical picture of coagulopathy associated with COVID-19 appears to be prothrombotic, and a high prevalence (25–42%) of venous thromboembolism (VTE), including deep vein thrombosis (DVT) and pulmonary embolism (PE), was observed in ICU patients with severe COVID-19 [29, 57, 73].

A multicenter prospective cohort study revealed that elevated d-dimer and fibrinogen levels at admission were present in 95% of COVID-19 patients in the ICU, and among 150 patients, 64 (42%) developed VTE including 25 cases of PE [57]. In addition, the study of 12 autopsy cases revealed DVT in seven of the 12 patients (58%), and PE was the direct cause of death in four patients [172]. It could be hypothesized that multi-organ injury is a result of microthrombus formation in the vital organs due to the prothrombotic state of COVID-19. It is uncertain whether the observed coagulopathy is caused by the virus or is secondary due to a cytokine storm. Early interventions such as anti-coagulant administration might be beneficial in severe COVID-19 patients [150], but this warrants further study.

### Kawasaki-Like Disease-Related SARS-CoV-2 Infection

According to cohort studies, most children who are infected with SARS-CoV-2 appear to have mild clinical symptoms and better prognoses compared to infected adults [48, 137]. However, shortly after the spread of SARS-CoV-2 infection was detected, the increased incidence of Kawasaki disease or Kawasaki-like disease was documented in western countries [55, 129, 161]. Eight of the ten pediatric patients identified had an antibody against SARS-CoV-2 [161]. Children diagnosed after the SARS-CoV-2 epidemic was identified were older and had a higher rate of cardiac dysfunction, and they showed features of macrophage activation syndrome (MAS), called “Kawasaki-like disease” [161]. The MAS criteria are validated for systemic juvenile idiopathic arthritis, but they are commonly used for other systemic auto-inflammatory diseases such as Kawasaki disease [41]. MAS is a form of cytokine storm in which clinical features (*i.e.*, high fever, lymphopenia, and high levels of transaminase, lactate dehydrogenase, d-dimer, and ferritin) are shared with severe COVID-19. Patients diagnosed with Kawasaki-like disease after the beginning of the SARS-CoV-2 epidemic showed a severe phenotype and required early interventions including steroid treatment [161] and intravenous immunoglobulin [55].

### Multi-organ Dysfunction/Injury and Outcomes

**Fig. 4:**
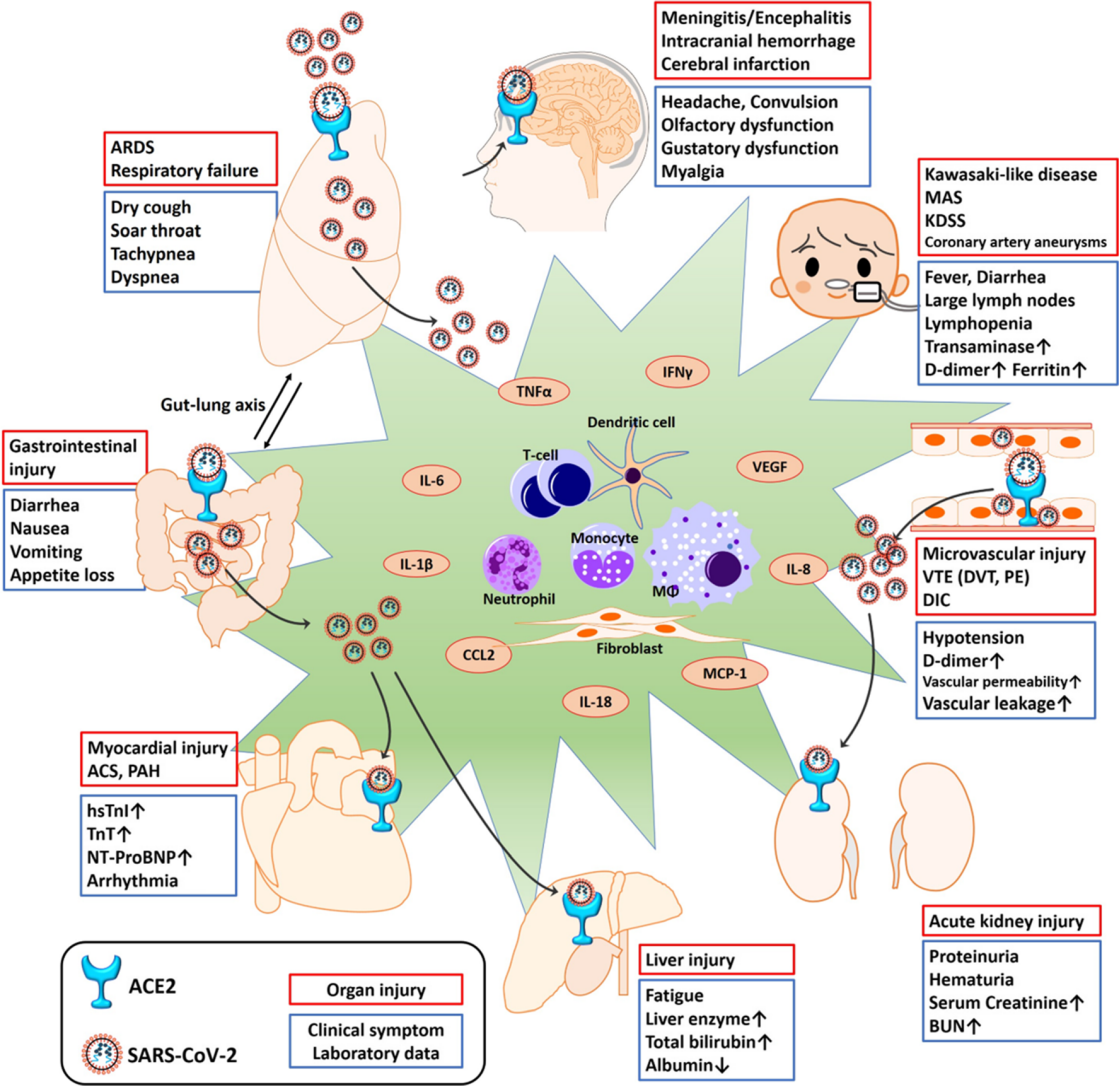
Clinical manifestations induced by SARS-CoV-2 infection. Organ dysfunction includes both direct cytotoxic effects of SARS-CoV-2 itself and cytokine-mediated damage. ACE2 was observed to be expressed in several types of tissue (including vessel endothelial and smooth muscle) and vital organs (lung, heart, intestine, brain, kidney, liver, *etc.*). The SARS-CoV-2 enters the host cells by binding to ACE2 and then directly damages the target organ. SARS-CoV-2 infection induces a release of proinflammatory cytokines (TNFα, IL-6 and others), resulting in injury to the target organ. ACS, acute coronary syndrome; ARDS, acute respiratory distress syndrome; BUN, blood urea nitrogen; CCL2, chemokine ligand 2; DIC, disseminated intravascular coagulation; DVT, deep vein thrombosis; hsTnI, high sensitive troponin I; IFNγ, interferon gamma; KDSS, Kawasaki disease shock syndrome; MAS, macrophage activation syndrome; MCP-1, monocyte chemoattractant protein 1; NT-ProBNP, N-terminal-pro hormone B-type natriuretic peptide; PAH, pulmonary artery hypertension; PE, pulmonary embolism; TnT, troponin T; VTE, venous thromboembolism.

Clinical observational studies revealed that the organ injuries discussed above are associated with death due to COVID-19. The reported prevalence of the injuries among patients with COVID-19 include ARDS (81–100%) [19, 183, 191, 197], cardiac injury (28–77%) [19, 183, 191, 197], AKI (38–50%) [183, 191, 197], liver dysfunction (25–28%) [183, 197], coagulopathy (50–71%) [151, 191], hypoxic encephalopathy (20%) [19], and gastrointestinal hemorrhage (6–8%) [183, 197]. The incidence of organ injury in the non-surviving patients was significantly higher than that in the surviving patients [19, 151, 183, 191, 197]. These organ injuries were independent predictors of death from COVID-19 [21, 139] (Fig. 4).

## FUTURE PERSPECTIVES ON THERAPEUTICS AND ONGOING STUDIES

### The Evidence About COVID-19 Treatment

As of the end of May 2020, almost 500 interventional studies were ongoing or planned across the world. There is only one double-blinded, full-powered randomized trial (RCT) about COVDI-19 treatment that has been completed and published: four clinical trials of remdesivir, and one controlled prospective study and four controlled clinical trials of chloroquine. The US Food and Drug Administration (FDA) has not approved any drugs for COVID-19 treatment as of this writing. There are also no US National Institutes of Health (NIH) treatment guidelines, due to the insufficient amount of clinical data that are necessary to determine a drug’s efficacy and safety.

Remdesivir, a viral RNA polymerase inhibitor, has been reported to inhibit SARS-CoV-2 polyproteolysis *in vitro* [191] and was administered as compassionate use in a case series [47] with clinical improvements. Four clinical trials of remdesivir were completed or the trials’ preliminary results were reported. The only double-blinded full-powered RCT to date is the Adaptive COVID-19 Treatment Trial (ACTT trial, *n* = 1063 patients, NCT04280705), which has shown that remdesivir accelerated recovery compared to the placebo (11 days *vs.* 15 days, *p* < 0.001, [6]) with a tentative finding of a lower mortality rate (8.0% *vs.* 11.6%, *p* = 0.059, press release). The subgroup analysis and the further outcome analysis with a larger patient number will be published eventually.

A comparison of remdesivir treatment (*n* = 155) and placebo (*n* = 78) for COVID-19 patients in China [21] (NCT04257656) was initiated for patients who exhibited severe disease within 14 days after symptom onset, but this double-blind placebo-controlled RCT was terminated early because there was no difference in clinical improvement between the remdesivir and placebo: hazard ratio (HR) 1.23, 95% confidence interval (CI), 0.87–1.75. Two SIMPLE trials conducted by the biopharmaceutical company Gilead (severe patients in NCT04292899 and moderate patients in NCT04292730) compared the clinical outcomes on day 14 between 5-day (*n* = 200) and 10-day (*n* = 197) remdesivir treatment starting within 4 days after the diagnosis among moderate or severe COVID-19 patients. The primary results showed no significant difference in outcomes or adverse events overall [46]. These trials are planning to recruit 6000 severe patients and 1600 moderate patients to be completed.

Lopinavir-ritonavir (L/R) is a human immunodeficiency virus (HIV) protease inhibitor. There is currently no large RCT testing the benefit and safety of L/R for COVID-19 patients. The latest open-label RCT with 199 severe patients revealed that compared to the control group, treatment with lopinavir (400 mg) and ritonavir (100 mg) did not boost the patients’ symptom recovery (HR 1.24, 95% CI 0.90–1.72) or their outcomes (mortality at day 28, L/R 19.2% *vs.* control 25%, 95% CI − 17.3–5.7) [17].

### Ongoing Clinical Trials Targeting ACE2

#### Endocytosis Inhibition: Chloroquine

Chloroquine (CQ) is a classic antimalarial and a potential antiviral agent [135]. *In vitro*, chloroquine effectively inhibited SARS-CoV-2 entry into cells (EC50 = 1.13 μM) [191], possibly by binding to the host respiratory cells, which would inhibit the attachment of the virus S1 protein to the host ACE2 on the host cells [36]. Five placebo-controlled clinical studies have described the outcomes of chloroquine treatment for COVID-19 patients.

A recent large placebo-controlled RCT with 14,888 COVID-19 patients indicated that hydroxychloroquine (HCQ) or CQ or in combination with a macrolide was related to worse in-hospital mortality [96]. In that RCT, 1868 patients received CQ alone, 3783 patients received CQ with a macrolide, 3016 patients received HCQ alone, and 6221 patients received HCQ with a macrolide; 81,144 patients served as the control group. The in-hospital mortality rates were higher in each treated group compared to the control after the cross-matching of the confounding factors: in-hospital mortality, control 9.3%; HCQ 18.0% (HR 1.335, 95% CI 1.223–1.457); HCQ + macrolide 23.8% (HR 1.447, 95% CI 1.368–1.531); CQ 16.4% (HR 1.365, 95% CI 1.218–1.531); and CQ + macrolide 22.2% (HR 1.368, 95% CI 1.273–1.469). Moreover, each treated group had ventricular arrhythmia during hospitalization compared to the control [96].

An interventional study of 181 COVID-19 patients (84 received CQ 600 mg/day *vs.* 97 without chloroquine) showed no improvement of symptoms, mortality, or transfer to the ICU at day 7 after hospitalization [90]. Another open-label RCT of 150 patients treated with HCQ showed no significant improvement in virus conversion (HCQ *vs.* control: 8 days *vs.* 7 days, HR 0.85, 95% CI 0.58–1.2; *p* = 0.34) or the symptom recovery time (HCQ *vs.* control: 19 days *vs.* 21 days, HR 1.01, 95% CI 0.59–1.74; *p* = 0.97) up to day 28 [152]. A small study of 62 patients in China indicated that CQ 400 mg/day might improve pneumonia in mild-moderate COVID-19, but a significant difference was not observed (ChiCTR2000029559) [172]. Another small controlled prospective study showed that compared to the control group (*n* = 20), HCQ (600 mg/day, *n* = 16) improved the virus conversion at day 3 and later (control *vs.* HCQ, 6% *vs.* 50%, *p* = 0.005) [42].

#### Cytokine Storm Blockades: Anti-TMPRSS2, Recombinant Human ACE2, ARB/ACE Inhibitors, JAK-STAT Inhibitor, Akt Pathway Inhibitor, and Anti-IL-6

As described above, virus infection *via* the host ACE2 induces the release of IL-6 *via* AT1Rs and the JAK-STAT and Akt pathways, leading to the amplification of the virus. As of the end of May 2020, there have been only retrospective studies of reagents used to treat SARS-CoV-2. Anti-TMPRSS2 agents can inhibit the cleavage of the S protein of this virus before its attachment. A high dose (100 mg/mL) of camostat mesylate was reported to inhibit the attachment of the S protein of SARS-CoV-2 to cells and that TMPRSS2 reduced the growth of SARS-CoV-2 *in vitro* [60].

One retrospective clinical study reported that anti-coagulant therapy with heparin improved the mortality rate among 449 severe COVID-19 patients with severe coagulopathy (d-dimer > 3.0 μg/mL, 32.8% *vs.* 52.4%, *p* = 0.017) [150]. Other studies showed that recombinant human ACE2 (rhACE2) was able to inhibit SARS-CoV-2 attachment to the host ACE2 by binding to the virus S protein [5], suppress viral growth efficiently *in vitro* [100], and improve severe lung inflammation *in vivo* [62]. The safety of rhACE2 has been confirmed by phase II/III studies with 39 ARDS patients (NCT01597635) [69].

Angiotensin receptor blockers (ARBs)/ACE inhibitors (ACE-Is) are widely used for the treatment of hypertension, which is commonly observed in COVID-19 patients [48]. There is no consensus as of the end of May 2020 regarding the use of ARBs/ACE-Is in COVID-19 patients, including ARBs (losartan, telmisartan) and ACE-Is (ramipril) due to insufficient clinical data about the adverse effects and outcomes. There have been several clinical studies of the relationships between ARB/ACE-I use and COVID-19; one case-controlled study and four retrospective studies.

The case-controlled study was of the ARB/ACE-I treatment of 6272 COVID-19 patients. The study results showed that ARB/ACE-I use did not change the patients’ overall outcome (adjusted odds ratio [OR] 0.95, 95% CI 0.86–1.05 for ARB, 0.96, 95% CI 0.87–1.07 for ACE-I) or the rate of critical or fatal disease (adjusted OR 0.83, 95% CI 0.63–1.10 for ARB, 0.91, 95% CI 0.69–1.21 for ACE-I) [91]. A large multinational retrospective study of 8910 patients indicated that the use of an ACE-I (*n* = 770, OR 0.33, 95% CI 0.20–0.54) or an ARB (*n* = 556, OR 1.23, 95% CI 0.87–1.74) had no effect on the likelihood of death from COVID-19 [95].

A large retrospective single-center study of 12,594 patients showed no association between ARB/ACE-I use and the severity/mortality of COVID-19 (24.7% *vs.* 24.8%, 95% CI − 3.5–3.5) [125], as did another retrospective study (severity 32.9% *vs.* 30.7%, *p* = 0.65, mortality 27.3% *vs.* 33.0%, *p* = 0.34) [84]. Two retrospective studies with > 100 hospitalized patients indicated that the use of an ARB/ACE-I had no correlation with clinical outcomes (HR 0.97, 95% CI 0.68–1.39; *p* = 0.88) [154] or the severity of COVID-19 symptoms [182].

IL-6 has been considered a key factor in the cytokine storm observed in COVID-19, and it is a potential therapeutic target. Tocilizumab is an anti-IL-6 monoclonal antibody with clinical evidence in rheumatoid arthritis treatment. A single-center study reported that 80–600 mg tocilizumab stabilized the symptoms in most of the 15 patients with moderate-to-severe COVID-19 [89]. JAK inhibitors, which were originally used as a treatment for myelofibrosis or polycythemia vera, act by a selective inhibition of JAK1/JAK2 [115]. *In vitro*, the JAK-STAT inhibitor baricitinib seemed to influence the viral entry and inflammation response in COVID-19 [126]. There are no reported clinical trials, retrospective studies, or case series concerning the effects of JAK-STAT inhibitors in COVDI-19 treatment.

### Future Perspectives

To date, there are no sufficient clinical data to suggest any effective treatments for COVID-19. It is necessary to gather more clinical data from controlled full-powered prospective studies. As listed on ClinicalTrials.gov., several hundred clinical trials of numerous medications are proceeding: anti-coagulants (nafamostat mesylate, camostat mesylate, heparin, enoxaparin, tinzaparin), ARBs/ACE-Is (losartan, telmisartan, ramipril), an anti-Ang2 agent (LY3127804), anti-mIL-6R drugs (sarilumab, siltuximab, tocilizumab), anti-IL-6 medications (clazakizumab, olokizumab, sirukumab), and JAK-STAT inhibitors (ruxolitinib, baricitinib, TD-0903). The therapeutic targets considered thus far are summarized in Figs. 3 and 4.

## CONCLUSION

This review has summarized the latest evidence regarding the mechanisms underlying COVID-19 and its associated multi-organ injuries and failure, plus the potential strategies for treatments to be studied/developed. Since SARS-CoV-2 binds to the host ACE2 with a subsequent release of proinflammatory cytokines, the primary infection and the characteristic symptoms can occur in the lungs and nasal airway where ACE2 and TMPRSS2 are strongly co-expressed, followed by vital organ injury due to a cytokine storm that is probably initiated by IL-6. Further clinical studies of the therapeutics for COVID-19 are urgently needed as antibody drugs and vaccines that can effectively cure the disease or tackle the SARS-CoV-2 pandemic are not yet available.
